# Advances and Perspectives for Genome Editing Tools of *Corynebacterium glutamicum*

**DOI:** 10.3389/fmicb.2021.654058

**Published:** 2021-04-07

**Authors:** Qingzhuo Wang, Jiao Zhang, Naief H. Al Makishah, Xiaoman Sun, Zhiqiang Wen, Yu Jiang, Sheng Yang

**Affiliations:** ^1^School of Food Science and Pharmaceutical Engineering, Nanjing Normal University, Nanjing, China; ^2^Key Laboratory of Synthetic Biology, CAS Center for Excellence in Molecular Plant Sciences, Shanghai Institute of Plant Physiology and Ecology, Chinese Academy of Sciences, Shanghai, China; ^3^University of Chinese Academy of Sciences, Beijing, China; ^4^Environmental Sciences Department, Faculty of Meteorology, Environment and Arid Land Agriculture, King Abdulaziz University, Jeddah, Saudi Arabia; ^5^School of Environmental and Biological Engineering, Nanjing University of Science & Technology, Nanjing, China; ^6^Huzhou Center of Industrial Biotechnology, Shanghai Institutes of Biological Sciences, Chinese Academy of Sciences, Shanghai, China

**Keywords:** *Corynebacterium*, molecular genetic modification, CRISPR/Cas system, genome editing, toolbox

## Abstract

*Corynebacterium glutamicum* has been considered a promising synthetic biological platform for biomanufacturing and bioremediation. However, there are still some challenges in genetic manipulation of *C. glutamicum*. Recently, more and more genetic parts or elements (replicons, promoters, reporter genes, and selectable markers) have been mined, characterized, and applied. In addition, continuous improvement of classic molecular genetic manipulation techniques, such as allelic exchange via single/double-crossover, nuclease-mediated site-specific recombination, RecT-mediated single-chain recombination, actinophages integrase-mediated integration, and transposition mutation, has accelerated the molecular study of *C. glutamicum.* More importantly, emerging gene editing tools based on the CRISPR/Cas system is revolutionarily rewriting the pattern of genetic manipulation technology development for *C. glutamicum*, which made gene reprogramming, such as insertion, deletion, replacement, and point mutation, much more efficient and simpler. This review summarized the recent progress in molecular genetic manipulation technology development of *C. glutamicum* and discussed the bottlenecks and perspectives for future research of *C. glutamicum* as a distinctive microbial chassis.

## Introduction

*Corynebacterium glutamicum* has been widely used in the food industry for amino acid production (Wendisch et al., 2016). It is also being considered as a promising general-purpose chassis strain for other high-value chemicals (Woo and Park, 2014; Heider and Wendisch, 2015; Becker et al., 2018), as well as an emerging heterologous protein expression host (Liu et al., 2016). However, there are some challenges in developing *C. glutamicum* as a synthetic biology platform (Woo and Park, 2014), especially in the aspect of genome editing tools, lagging far behind *Escherichia coli*.

The genetic modification of *C. glutamicum* can be traced back to 1984 (Ozaki et al., 1984), but the development and application of genetic manipulation technology are progressing slowly (Nesvera and Patek, 2011; Suzuki and Inui, 2013; Yang et al., 2020), which may be attributed to the fact that *C. glutamicum* is a type of Gram-positive actinomyces with high GC content in the genome (Ikeda and Nakagawa, 2003). Unusual cell wall together with deficient homologous recombination (HR) (for DNA repair) of *C. glutamicum* results in extremely low efficiency in shuttle plasmid transformation and subsequent gene editing (Nesvera and Patek, 2011; Ruan et al., 2015; Yang et al., 2020). In the post-genomic era of *C. glutamicum* (Kalinowski et al., 2003; Lv et al., 2011, 2012), genomics and transcriptomics have promoted the mining and characterization of synthetic biological elements (such as promoters, replicons, and selectable markers) to a certain extent (Tauch et al., 2003; Nesvera et al., 2012; Patek et al., 2013; Rytter et al., 2014; Shang et al., 2018). More and more genetic manipulation tools have been applied in *C. glutamicum* (Nesvera and Patek, 2011; Suzuki and Inui, 2013), including type strain ATCC 13032 and no-model industrial strains such as *Brevibacterium flavum* and *Corynebacterium crenatum* (Xu et al., 2010; Shu et al., 2018). Most importantly, the gene editing technology mediated by CRISPR/Cas system has been successfully developed in *C. glutamicum*, revolutionizing the study of genetic manipulation technology (Jiang et al., 2017).

Here, we reviewed recent advances in genome editing technology of *C. glutamicum*, as summarized in Table 1, with a special focus on the CRISPR/Cas system. Technical bottlenecks and future development trends are also discussed.

**TABLE 1:** Comparison of different genetic tools applicable in *C. glutamicum.*

**Genome editing tools**	**Principles or outcome(s)**	**Advantages**	**Putative drawbacks**	**References**
Allelic exchange	HR (homologous recombination)-mediated in-frame deletion or insertion	Versatile and broadly applicable	Limited to low HR efficiency	Niebisch and Bott, 2001; Unthan et al., 2015; Baumgart et al., 2016; Baumgart et al., 2018
Counter-marker-assisted allelic exchange	Marker-mediated conditional lethality to retain mutants with second crossover	Filtering out false positives to reduce workload	Failing to stimulate HR	Niebisch and Bott, 2001; Kim et al., 2011; Ma et al., 2015
Cre-loxP or I-SceI system-assisted allelic exchange	DNA cleavage by Cre or I-SceI to accelerate second crossover	Filtering out false positives to reduce workload; stimulating HR	Remaining recognition site may interference next round of operation	Suzuki et al., 2005b; Suzuki et al., 2005d; Ma et al., 2015; Huang et al., 2017; Zhan et al., 2019; Luo et al., 2020; Marques et al., 2020; Wu et al., 2020
RecET/ssDNA(dsDNA)-mediated recombination	RecT recombination system-mediated HR	Independent of host recombination system; straightforward procedure	Limited to RecET expression and ssDNA/ds DNA transformation efficiency	Binder et al., 2013; Krylov et al., 2014; Huang et al., 2017; Su et al., 2018; Luo et al., 2020
CRISPR/Cas9	Cas9-mediated DSB to stimulate DNA repair	Broadly applicable and function diversity	Toxicity of DSB; limited to host DNA repair capability	Liu et al., 2017; Peng et al., 2017; Coates et al., 2019
CRISPR/Cas9 + RecT/ssDNA	Cas9-mediated DSB and RecT recombination system mediated HR	Enhanced recombination efficiency	Limited to RecT expression and ssDNA transformation efficiency	Cho et al., 2017; Liu et al., 2018; Wang B. et al., 2018
CRISPR/dCas9	Steric hindrance effect of dCas9 to repress transcription	Fine transcription level regulation of any given gene	Dependent on sgRNA and target gene	Cleto et al., 2016; Lee et al., 2018; Yoon and Woo, 2018; Gauttam et al., 2019
CRISPR/Cpf1	Cas9-mediated DSB to stimulate DNA repair	Decreased toxicity; multiple sites editing; broadly applicable	Toxicity of DSB; limited to host DNA repair capability	Jiang et al., 2017; Krumbach et al., 2019; Zhang et al., 2019; Dong et al., 2020; Li M. et al., 2020
CRISPR/Cpf1 + RecT/ssDNA	Cas9-mediated DSB and RecT recombination system mediated HR	Enhanced recombination efficiency and multiple sites editing	Limited to RecT expression and ssDNA transformation efficiency	Jiang et al., 2017; Wang et al., 2019b; Zhang J. et al., 2020; Zhao et al., 2020
CRISPR/dCpf1	Steric hindrance effect of dCpf1 to repress transcription	Fine transcription level regulation of any given gene	Dependent on sgRNA and target gene	Li M. et al., 2020
Cytosine base editor (CBE)	Activation-induced cytidine deaminase (AID) and CRISPR/dCas9 convert C to T in editing window	High efficiency and multiple sites editing	Limited base transition capability	Wang Y. et al., 2018; Deng et al., 2020; Li J. et al., 2020
Adenine base editor (ABE)	tRNA adenosine deaminase and CRISPR/dCas9 convert A to G in editing window	High efficiency and multiple sites editing	Limited base transition capability	Wang et al., 2019c; Deng et al., 2020
TadA-dCas9-AID	Combination of CBE and ABE	Bi-directional base conversion to achieve C–T, C–G and A–G conversion	Limited base transition capability	Deng et al., 2020
Base editor (BE3)	Cytidine deaminase and uracil DNA glycosylase inhibitor; converting specific C⋅G nucleotide base pairs to T⋅A	High efficiency and multiple sites editing	Limited base transition capability	Huang et al., 2020
MACBETH	Robotic system-assisted multiplex automated base editing	Automated, ultra-high-throughput multiple sites editing	Limited base transition capability	Wang Y. et al., 2018
Actinophages integrase mediated integration	TP901-1, ϕC31 or ϕBT1 integrase mediated integration	Site-directed integration of long DNA fragment	Attachment sites need to be installed in advance	Shen et al., 2017; Marques et al., 2020
Transposon	Random transposon disruption or inactivation	Easy to construct single-gene disruptant mutant library	Inaccurate genome editing	Vertes et al., 1994; Inui et al., 2005; Suzuki et al., 2006; Gorshkova et al., 2018
Transposon + Cre-loxP system	Random long or short DNA fragments deletion	Easy to construct reduced genome mutant library	Inaccurate genome reducing	Tsuge et al., 2007

## Classic Allelic-Exchange-Based Genome Editing Tools

**FIGURE 1:**
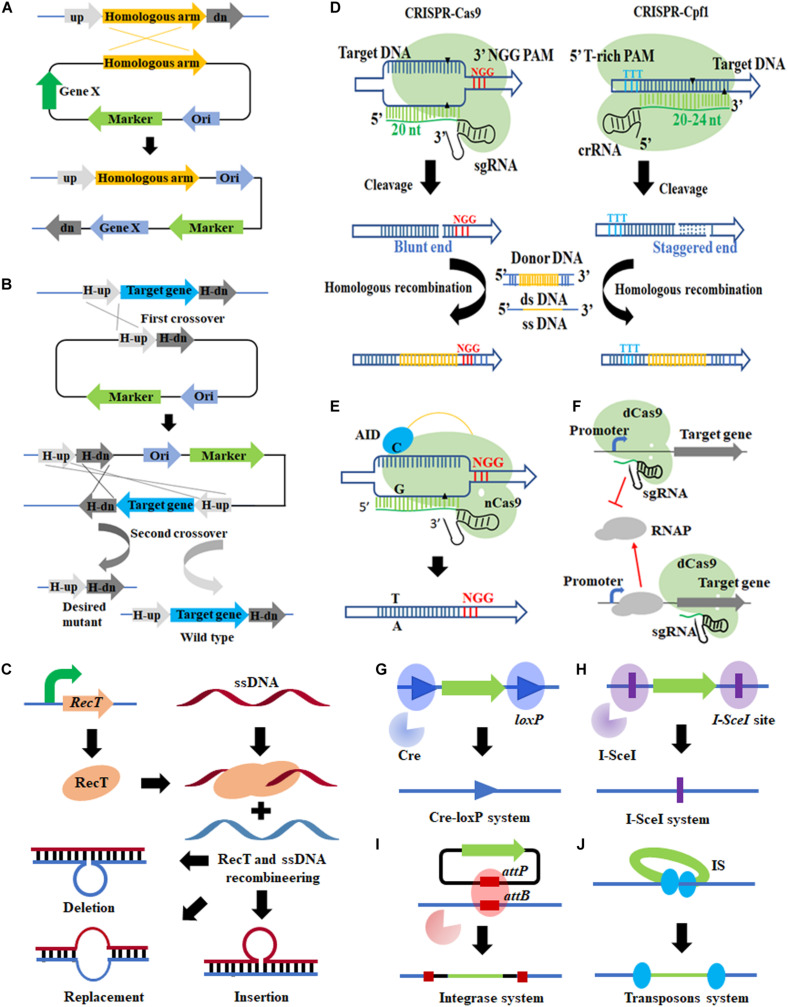
Genome editing tools applicable in *Corynebacterium glutamicum.* **(A,B)** Allelic exchange-based tools (single and double crossover); **(C)** RecT-ssDNA-mediated recombination; **(D)** CRISPR/Cas system; **(E)** CRISPR/Cas system-assisted base editing system; **(F)** CRISPR/Cas system-assisted transcription regulation; **(G)** Cre-loxP system; **(H)** I-SceI system; **(I)** integrase-mediated site-specific integration; **(J)** transposon.

Since *C. glutamicum* can hardly repair DNA through Non-Homologous End Joining (NHEJ), allelic exchange based on HR is the most commonly used genetic manipulation tool (Suzuki and Inui, 2013; Yang et al., 2020). In *C. glutamicum*, both suicide plasmid and replicable plasmid can be used for allelic exchange (Wang et al., 2019a; Wu et al., 2020). Allelic exchange could be achieved by single crossover and double crossover, the results of which vary dependent on the characteristic of homology arms (Figures 1A,B).

In *C. glutamicum*, genetic tools based on allelic exchange can basically implement genetic manipulation such as insertion, substitution, deletion, and point mutation (Nesvera and Patek, 2011; Suzuki and Inui, 2013; Yang et al., 2020) and have been widely used in metabolic engineering and chassis development (Woo and Park, 2014). However, there are some drawbacks for allelic exchange. For example, it usually takes a long period (about 8 days) to complete one round of gene editing. Besides, the low efficiency of the second single crossover prevents the desired mutant to be obtained, even after a large number of colony PCR screening (Wen et al., 2020). To ensure the availability of desired mutant strains, counter-selectable markers and nuclease systems are introduced.

*SacB* gene encoding a levansucrase, which can convert sucrose into a toxic metabolite, is the most commonly used counter-selectable marker in *C. glutamicum*. Schäfer et al. (1994) successfully deleted the hom-*thrB* gene of *C. glutamicum* by SacB-assisted allelic exchange. In later studies, the streptomycin-sensitive gene *rspl* and 5-fluorouracil-lethal gene *upp* were also introduced as negative markers in *C. glutamicum* (Kim et al., 2011; Ma et al., 2015). Screening marker-mediated conditional lethality can help filter out strains that have not undergone the secondary crossover, because only strains that have lost the lethal gene through the second crossover can survive. Therefore, the screening workload is drastically reduced. However, it does not improve the efficiency of HR.

In *C. glutamicum*, nuclease systems such as Cre-loxP and I-SceI system (Yang et al., 2020) have been introduced to force the host to activate a second crossover (by specifically cutting DNA) to survive (Zhang et al., 2015). It not only filters out the transformants that have not undergone the second crossover but also stimulates recombination. However, low-efficiency DNA repair still hinders the acquisition of desired transformants. Therefore, the RecT recombinase system was employed. RecT is a single-stranded DNA annealing protein (SSAPs) (Zhang et al., 1998), which can mediate binding of template DNA strand and homologous DNA by annealing, to realize subsequent exchange and invasion. Accordingly, artificially synthesized ssDNA substrates can effectively achieve site-directed mutagenesis, insertion, and deletion, through recombination (Figure 1C).

RecT-mediated ssDNA single-stranded recombination does not rely on the RecA recombination system of the bacteria, but relies on the RecT recombination system encoded by the *recT* gene from the prophage Rac (Zhang et al., 1998). Compared with the natural recombination system in hosts, it is easier to operate and is not affected by DNA sequence and length, which can achieve high-efficiency recombination using even very short homologous DNA sequences as substrates (Murphy et al., 2000; Sawitzke et al., 2011). Binder et al. (2013) introduced RecT recombinase into *C. glutamicum* for the first time. In later studies, Krylov et al. (2014) and Wu et al. (2020) optimized the ssDNA chain length, concentration, base modification, and DNA strand tendency (leading or lagging strand), which further improved the recombination efficiency of ssDNA in *C. glutamicum*. The exonuclease–recombinase pair RecE + RecT (RecET) has also be adapted to promote dsDNA recombination. Recently, Huang et al. (2017) reported an effective and sequential deletion method based on RecET and Cre/loxP system, which has been successfully applied for L-leucine production in *C. glutamicum* (Luo et al., 2020).

Although RecT/ssDNA- or RecET/dsDNA-mediated recombineering simplifies the operation of genome editing, only one gene can be edited at one round in *C. glutamicum*. By contrast, in *E. coli*, oligonucleotide-mediated multiple-site editing of the genome has been successfully applied for over 10 years (Wang et al., 2009; Isaacs et al., 2011). It implied that ssDNA/dsDNA electroporation efficiency and the expression level of the RecT/RecET in *C. glutamicum* need to be further optimized.

## Revolutionary CRISPR/Cas-Based Genome Editing Tools

The CRISPR/Cas system has achieved great success in various prokaryotic and eukaryotic microorganisms and is regarded as a revolutionary gene manipulation technology (Jinek et al., 2012; Cong et al., 2013). A lot of effort has been paid to introduce the CRISPR/Cas system into *C. glutamicum*, but progress was not smooth initially, because *C. glutamicum* cannot tolerate the toxicity of Cas9 expression (Cho et al., 2017; Jiang et al., 2017). This explains why CRISPR interference (CRISPRi) mediated by CRISPR/dCas9 [dead Cas9, harboring D10A and H840A mutations in Cas9, no nuclease activity (Bikard et al., 2013)], but not CRISPR/Cas9-based genome editing, was first applied to *C. glutamicum* (Cleto et al., 2016).

CRISPRi can be used to regulate the transcriptional level of any given gene by steric hindrance effect (Figure 1F) and is especially suitable to down-regulate essential genes because they cannot be inactivated directly (Wen et al., 2017). In *C. glutamicum*, CRISPR/dCas9-mediated single-gene transcriptional repression (Cleto et al., 2016; Lee et al., 2018; Yoon and Woo, 2018; Gauttam et al., 2019) and CRISPR-dCpf1-mediated down-regulation of multiple genes have been achieved (Liu et al., 2019; Li M. et al., 2020), but hardly no study about transcriptional activation has been reported.

As for genome editing, after observing lethality of Cas9 expression to *C. glutamicum*, Jiang et al. (2017) developed a gene editing tool based on *Francisella novicida* (Fn) CRISPR/Cpf1 (Figure 1D), in which DSB created by Cpf1 (a staggered end) can be repaired by DNA templates. When ssDNA and RecT recombination systems were introduced, more types of gene editing including gene deletion/insertion/point mutation were realized (Jiang et al., 2017). It represented a milestone in gene editing tools development of *C. glutamicum* and was successfully applied in six other industrial *Corynebacterium* strains.

Immediately after this work, breakthroughs in CRISPR/Cas9 system development were achieved. Cho et al. (2017) found that the codon optimization of the Cas9 gene reduced the toxicity of Cas9 expression; in addition, when RecT and ssDNA recombineering were employed to further facilitate recombination at the target loci, genome editing based on the CRISPR/Cas9 system in *C. glutamicum* was realized for the first time. In parallel studies, controlling the expression of Cas9 under an inducible promoter also achieved the goal of reducing toxicity (Liu et al., 2017; Peng et al., 2017).

The CRISPR/Cas system was subsequently optimized in the aspects of Cas9 expression stability (Wang B. et al., 2018), the convenience of curing Cas9 plasmids (Cho et al., 2017), transformation efficiency of Cas9 plasmids (Coates et al., 2019), crRNA delivery vector design (Krumbach et al., 2019), protospacer adjacent motif (PAM) sequence, the length of the spacer sequence, and the type of repair template (Wang et al., 2019c; Zhang et al., 2019), among others. Moreover, counter-selectable markers (Zhang J. et al., 2020) and ssDNA-RecT recombination engineering (Liu et al., 2018; Wang B. et al., 2018; Zhao et al., 2020) have also been introduced to further optimize the gene editing system. The application of the CRISPR/Cas system has been expanded from single gene editing to multiplex gene editing and large DNA fragment deletion (Liu et al., 2017; Wang B. et al., 2018; Zhao et al., 2020). However, it is still conditioned to the inefficient HR of *C. glutamicum*.

Base editing can create a missense mutation or null mutation in a gene via base substitution without introducing a DSB (Komor et al., 2016; Nishida et al., 2016), which is especially suitable for strains with inefficient HR (like *C. glutamicum*), and has attracted increasing attention (Wen et al., 2020). Wang Y. et al. (2018) developed a cytosine base editor (CBE) applicable in *C. glutamicum* based on activation-induced cytidine deaminase (AID) and the CRISPR/Cas9 system (Figure 1E), which can efficiently achieve C–T conversion with efficiencies up to 100%, 87.2%, and 23.3% for single-, double-, and triple-locus editing, respectively. In subsequent work, they fused tRNA adenosine deaminase from *E. coli* (TadA) with different Cas9 variants to construct different adenine base editors (ABEs), which can convert specific A⋅T nucleotide base pairs in the CRISPR-Cas9 targeting window sequence to G⋅C (Wang et al., 2019c). By combining the above CBE and ABE tools in one system, Deng et al. (2020) developed a bi-directional base conversion tool TadA-dCas9-AID, which achieved the base conversion of C–T, C–G, and A–G in the editing window. Most recently, Huang et al. developed a BE3 Cytidine Base Editor by fusing the cytidine deaminase (rat Apobec1), nCas9, and uracil DNA glycosylase inhibitor (UGI). It can convert C to T with a conversion efficiency up to 90% (Huang et al., 2020), which provided more tools for base editing.

Parallel to base editing tools development to explore different base transition capability, the system optimization has also made progress. Wang et al. found that some Cas9 variants can accept different PAM sequences, which increased their genome-targeting scope for base editing. Besides, base editing window was expanded from 5 to 7 bp when truncated or extended guide RNAs were adapted (Wang et al., 2019c). They also provided an online tool (gBIG^1^) for designing guide RNAs for base editing-mediated inactivation (Wang et al., 2019c). It is particularly exciting that an integrated robotic system-assisted automation base editing platform based on MACBETH was constructed (Wang Y. et al., 2018), which represented a new trend in future studies.

CRISPR/Cas system-based genome and base editing tools have brought the development of genetic manipulation technology into a new era due to its multiple functions, higher efficiency, shorter cycle, and more sophisticated modification over the traditional allelic exchange (Cho et al., 2017; Jiang et al., 2017; Wang Y. et al., 2018). Currently, the CRISPR/Cas system is generally preferred and increasingly applied in strain breeding (Bott and Eggeling, 2017), including rapid identification of unknown genes (Lee et al., 2018), complicated metabolic engineering (Zhang J. et al., 2020), and rational genome evolution (Zhao et al., 2020).

## Indispensable HR-Independent Genome Editing Tools

There are some HR-independent genome editing tools applicable in *C. glutamicum*, such as the aforementioned Cre-loxP and I-SceI systems. Nuclease Cre mediates intramolecular recombination of two loxP sites (Figure 1G), so that the DNA sequence between the loxP sites is deleted or rearranged, leaving a loxP site in chromosome (Suzuki et al., 2005a,d). Suzuki et al. (2005c; 2005d) realized large fragment deletion and genome rearrangement in *C. glutamicum* using the Cre-loxP system. A total of 11 distinct genomic regions (up to 250 kb, 7.5% of the genome) were successfully deleted. A putative problem is that the loxP sites remaining in chromosome may interfere with subsequent rounds of Cre/loxP recombination (Suzuki et al., 2005a). To avoid that, a pair of mutant lox sites (lox66 and lox71) was introduced to replace the loxP site. The lox72 site, generated from Cre, caused site-specific recombination of lox66 and lox71 and cannot be recognized by Cre, which facilitated continuous Cre-lox recombination (Suzuki et al., 2005a; Hu et al., 2014).

As for the I-SceI system (Figure 1H), it consists of a homing endonuclease I-SceI and an 18-bp specific sequence (5′-TAGGGATAACAGGGTAAT-3′) (Zhang et al., 2015). The system has been adapted in *C. glutamicum* for genes knock-out and knock-in (Suzuki et al., 2005b; Ma et al., 2015; Wu et al., 2020). In addition, it is often used in conjunction with counter-selectable markers such as *SacB*, *Upp*, and the Cre-loxP system (Suzuki et al., 2005d; Ma et al., 2015; Wu et al., 2020).

It should be noted that the specific recognition sequence of the nuclease is difficult to customize, and the recognition site of the recombinase must be introduced into the chromosome in advance (Nesvera and Patek, 2011; Suzuki and Inui, 2013; Yang et al., 2020). It explained why site-specific recombination tools are usually used in combination with allelic exchange-based tools.

Different from Cre-loxP and I-SceI systems, transposon is a simple tool that can perform genome editing independently (Figure 1J). It can cause interruption or inactivation of some genes by random transposon insertion (Alain et al., 1994). Many transposable elements have been identified and used in *C. glutamicum*, such as IS31831 (Vertes et al., 1994), miniTn31831 (Alain et al., 1994), Tn14751 (Inui et al., 2005), IS1249 (Tauch et al., 1995), Tn5 (Suzuki et al., 2006), and mini-Mu (Gorshkova et al., 2018). These transposons have different transposition efficiency, sequence preference (AT-rich regions or GC-rich regions preference), and cargo delivery capacity. The combined application of different transposable elements can make up for each other’s deficiencies, thereby identifying more genes with unknown functions. For example, a combination of the miniTn31831 and Tn5 transposome systems successfully constructed a pool of 13,000 transposon mutants, equal to a library of 2300 different single-gene disruptant mutants, covering 75% of genes in *C. glutamicum* (Suzuki et al., 2006).

Transposon can be used not only for random knockout of single gene but also for random deletion of chromosome fragments when it is combined with nuclease systems (Goryshin et al., 2003; Tsuge et al., 2007). Random segment deletion based on IS31831 and Cre/loxP excision system has been applied for genome reduction of *C. glutamicum* by random deletion of DNA fragments (Tsuge et al., 2007). Compared with conventional strategies (genomic analysis combined with precise deletion) (Baumgart et al., 2013, 2016, 2018; Unthan et al., 2015), this strategy has been considered as a faster way to create a minimum bacterial genome (Suzuki and Inui, 2013).

Unfortunately, transposons are rarely used for random integration of heterogenous genes, because the length of cargo fragments is usually limited (Gorshkova et al., 2018). By contrast, integrase-mediated site-directed integration of heterologous genes can integrate fragments up to tens of kb (Figure 1I) (Huang et al., 2019). Shen et al. (2017) employed a phage integrase TP901-1-mediated chromosomal integration method in *C. glutamicum*, which realized the integration of two reporter genes, implying good application potential. In another study, Marques et al. (2020) developed two markerless integrative systems, respectively, based on actinophage ϕC31 and ϕBT1 for stable inheritance of the introduced genetic traits. Similar to the Cre-loxP and I-SceI system, the prerequisite for integrase-mediated site-directed integration is that the attB site has been integrated into the chromosome by allelic exchange in advance (Shen et al., 2017; Marques et al., 2020). To realize one-step site-directed integration, Strecker et al. (2019) and Klompe et al. (2019), respectively, developed CRISPR-associated transposon-mediated RNA-guided programmable DNA integration methods in *E. coli*. These methods do not rely on HR and have achieved multi-site and multi-copy integration of heterologous genes in *E. coli* and *Tatumella citrea* (Vo et al., 2020; Zhang Y. et al., 2020). It is expected to be introduced into *C. glutamicum* for high-efficiency, multiplexed chromosome integration.

Table 1 summarizes and compares the principles, effects, advantages, and putative drawbacks of various genetic manipulation tools in *C. glutamicum*. According to the table, the CRISPR/Cas system has obvious advantages over other methods but is far from perfect. Classic tools such as counter-selectable markers, ssDNA-RecT recombineering, transposons, and nuclease could be employed in the CRISPR/Cas system to further improve efficiency and expand functions. The combination of different genetic manipulation tools to achieve new editing purposes has become a trend (Suzuki and Inui, 2013; Yang et al., 2020). Besides, many efforts have been paid to overcome barriers to introduce these tools to non-model *Corynebacterium* strains (Jiang et al., 2017; Coates et al., 2019).

Synthetic biology is profoundly rewriting the development pattern of genetic modification of *C. glutamicum*. Artificial intelligence-assisted massive omics data mining may greatly enrich the genetic element library of *C. glutamicum*; advanced models or algorithms could rationally guide chassis cells design; coupled with a new generation of high-throughput, automated biological casting platform, they should enable the future development of more effective *C. glutamicum*.

## Author Contributions

QW, XS, and ZW conceived the project and wrote the manuscript. All authors participated in the discussion, revised the manuscript, and approved the final manuscript.

## Conflict of Interest

The authors declare that the research was conducted in the absence of any commercial or financial relationships that could be construed as a potential conflict of interest.

## Footnotes

1 http://www.ibiodesign.net/gBIG
